# *Angiostrongylus cantonensis* Rat Lungworm Detected in Rats, Madagascar, 2022–2023

**DOI:** 10.3201/eid3207.260142

**Published:** 2026-07

**Authors:** Santatriniaina F. Randrianarisoa, Elise N. Paietta, Rachel A. Johnston, Tahina T. Razanamahenina, Antsa Ramboninarimalala, Toussaint G. Raherinirina, Laurent Raveloson, Nina L. Finley, Eric Baitchman, Arvind Varsani, Fidisoa T. Rasambainarivo

**Affiliations:** Mahaliana Labs, Antananarivo, Madagascar (S.F. Randrianarisoa, F.T. Rasambainarivo); Duke University, Durham, North Carolina, USA (E.N. Paietta); Arizona State University, Tempe, Arizona, USA (E.N. Paietta, A. Varsani); Broad Institute, Cambridge, Massachusetts, USA (R.A. Johnston); Zoo New England, Boston, Massachusetts, USA (R.A. Johnston, E. Baitchman); University of Antananarivo, Antananarivo (T.T. Razanamahenina, A. Ramboninarimalala); Centre ValBio, Ranomafana, Madagascar (T.G. Raherinirina); Health In Harmony-Fahasalamana Mirindra, Farafangana, Madagascar (L. Raveloson, N.L. Finley); London School of Hygiene and Tropical Medicine, London, UK (N.L. Finley); University of Cape Town, Cape Town, South Africa (A. Varsani); East Carolina University, Greenville, North Carolina, USA (F.T. Rasambainarivo)

**Keywords:** *Angiostrongylus cantonensis*, Rat Lungworm, *Rattus rattus*, zoonotic parasite, parasites, zoonoses, Mitogenome, Madagascar

## Abstract

*Angiostrongylus cantonensis*, the rat lungworm, is a zoonotic parasite that causes eosinophilic meningitis in humans; the parasite is maintained in rat definitive hosts and transmitted through gastropod intermediate hosts. We report *A. cantonensis* prevalence and mitochondrial genome from oral swab specimens from rats in Madagascar, supporting swabs for noninvasive detection of this parasite.

*Angiostrongylus cantonensis*, the rat lungworm, is a zoonotic nematode and a leading cause of eosinophilic meningitis in humans and other mammals (*1*). Originally described in rats in China in 1935, *A. cantonensis* lungworm is now reported in >30 countries globally (*2*). Distribution has expanded because of globalization, human activity, and range expansion of definitive and intermediate hosts (*2*,*3*).

Adult *A. cantonensis* worms inhabit the pulmonary arteries of rats (definitive hosts), including black rats (*Rattus rattus*) and brown rats (*Rattus norvegicus*), where eggs hatch into first-stage larvae shed in feces. Those larvae infect intermediate hosts such as snails and slugs, where they develop into third-stage larvae and can be transmitted via paratenic hosts (including reptiles and amphibians), where larvae can persist for extended periods, and through transient hosts (e.g., crustaceans and insects), where larvae survive for only a short time (*4*). Rats acquire infection by ingesting those hosts, completing the life cycle (*5*). Humans are infected accidentally through consumption of raw or undercooked intermediate or paratenic hosts or via contaminated food and water (*6*). After ingestion, the larvae migrate from the intestines to the brain, causing meningitis and neurologic complications (*4*,*7*).

Madagascar is one of the world’s most expansive biodiversity hotspots, where humans and an exceptional diversity of endemic wildlife frequently interact with nonnative animals across the island (*8*). Although *A. cantonensis* worms have been reported in the region (*9*), genomic data and epidemiologic insights remain limited. In this study, we used metagenomic sequencing of oral swabs to characterize mitochondrial genomes and estimate prevalence of *A. cantonensis* lungworm in black rats from southeastern Madagascar.

## The Study

**Figure 1:**
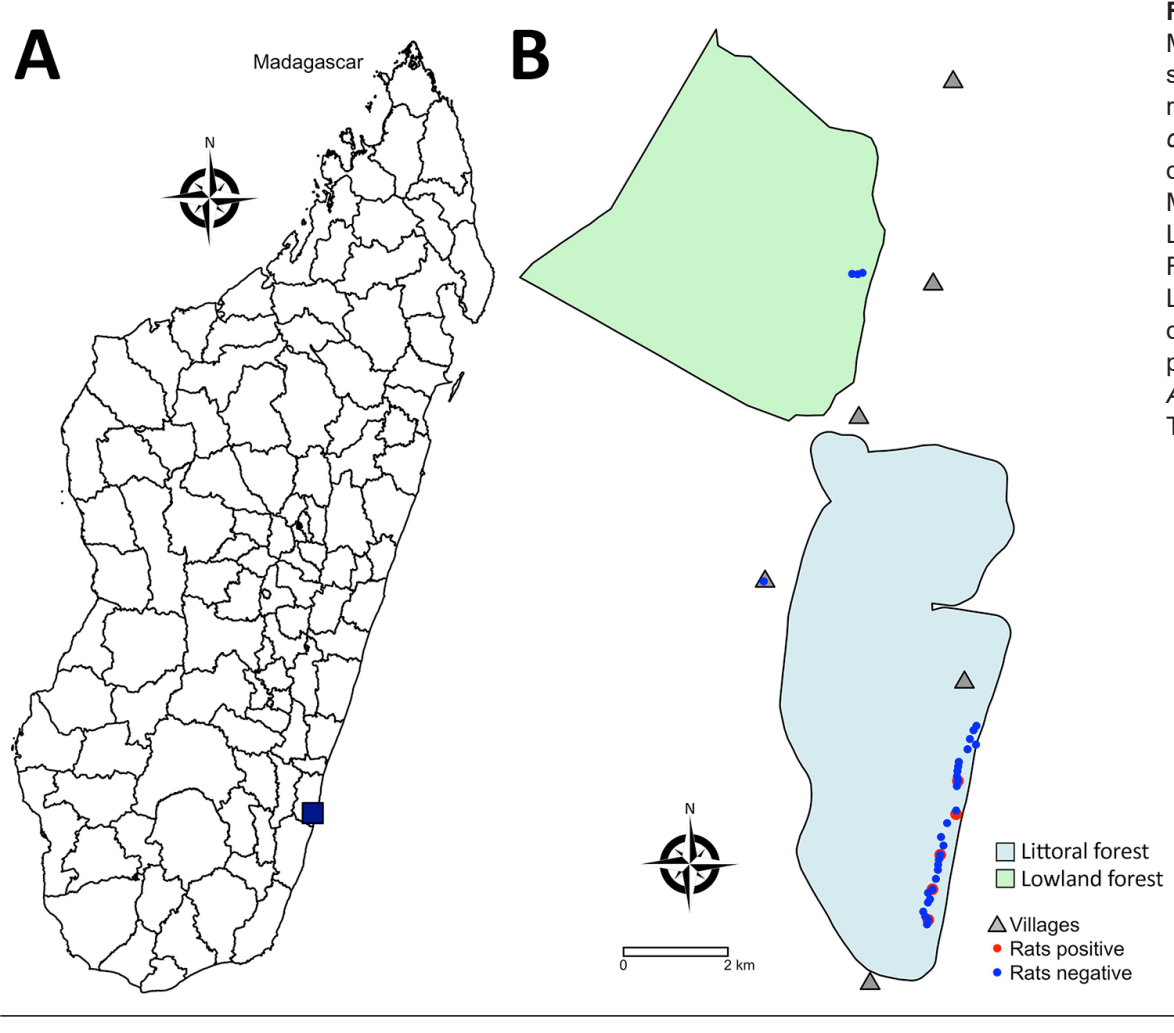
Geographic location of Manombo Special Reserve and spatial distribution of sampled rats in a study of *Angiostrongylus cantonensis* rat lungworm detection in rats in southeastern Madagascar, 2022–2023. A) Location of Manombo Special Reserve (black square). B) Locations of sampled rats; red dots indicate *A. cantonensis*–positive rats; blue dots indicate *A. cantonensis*–negative rats. Triangles indicate villages.

As part of a broader One Health study (*10*), we collected oral swab samples from 125 wild black rats that were captured, anesthetized, and euthanized during October 2022 and July 2023 in the Manombo Special Reserve (MSR), southeastern Madagascar (Figure 1, panels A, B). The samples were collected in 3 different habitats: littoral forest (n = 76 samples), lowland rainforest (n = 22 samples), and village-edge (n = 27 samples). 

We conducted DNA extraction by using the Roche HighPure Viral Nucleic Acid Kit (Roche, https://www.roche.com). We followed our previously published pipeline for library preparation, sequencing, bioinformatic processing, quality trimming, de novo assembly, and contig screening by using standard tools and the National Center for Biotechnology Information (NCBI) mitochondrial RefSeq database (*11*). We annotated mitochondrial genomes by using reference genomes of *A. cantonensis* (NCBI Sequence Read Archive accession no. GQ398121) with manual curation.

For phylogenetic analysis, we aligned complete mitochondrial genomes with publicly available *A. cantonensis* sequences. We constructed a maximum-likelihood tree by using IQ-Tree 2 (*12*) to determine the genetic relationships of our samples and haplotype placement.

Because metagenome-assembled *A. cantonensis* mitogenomes were highly similar, we mapped raw reads to a representative mitogenome by using CoverM (*13*) with a minimum read identity of 95%. We used a read coverage threshold of >75% to determine presence or absence of *A. cantonensis* genomes in each sample, providing a high-confidence proxy for infection status and calculated prevalence accordingly. To identify predictors of infection, we performed a generalized linear model in R version 4.4.1 (The R Project for Statistical Computing, https://www.r-project.org), testing age, sex, location, body condition, weight, and year of sampling.

All procedures were approved by Duke University Institutional Animal Care and Use committee (approval no. A075‐23‐03), Zoo New England ethics board, and the Madagascar Ministry of the Environment (approval nos. 286/22 and 215/23/MEDD/SG/DGGE/DAPRNE/SCBE.Re). We deposited all raw sequencing data in the NCBI Sequence Read Archive. Taxonomic classification revealed that 26.26%–51.43% of reads per sample were assigned to known taxa, including bacteria (15.01%–47.72%), eukaryotes (0.53%–4.62%), and viruses (0.04%–18.78%). Viral sequences mainly comprised anelloviruses and bacteriophages (*10*).

**Figure 2:**
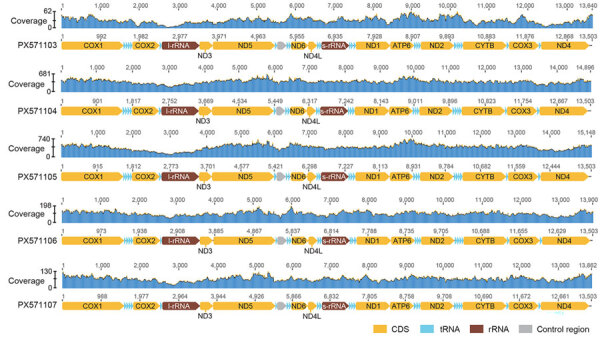
Mitochondrial genome organization and sequence coverage maps of *Angiostrongylus cantonensis* rat lungworm from black rat oral swab specimens in southeastern Madagascar, 2022–2023.

**Figure 3:**
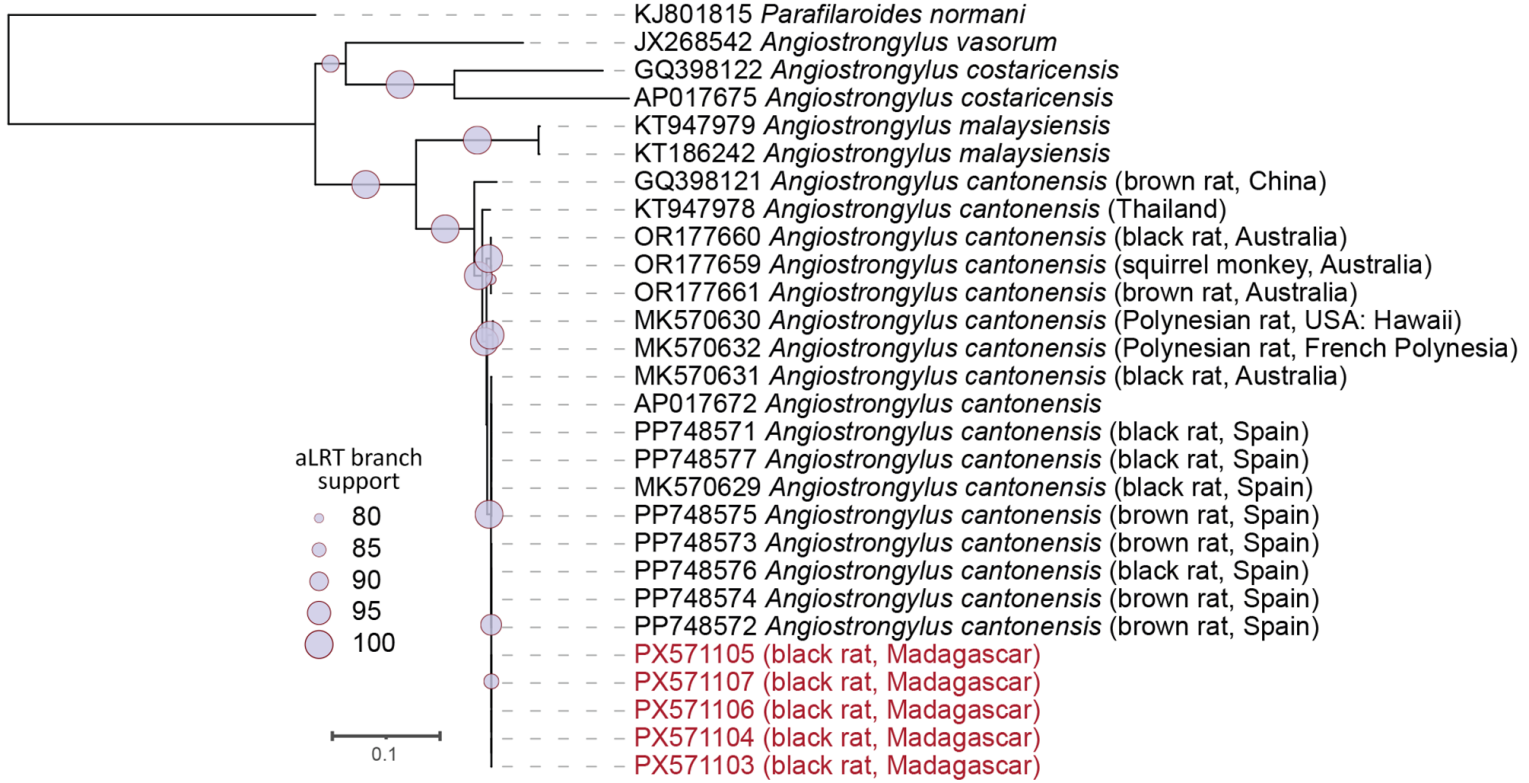
Whole-mitogenome maximum likelihood phylogenetic tree of complete sequences available for *Angiostrongylus cantonensis* rat lungworm and 3 related *Angiostrongylus* species along with the sequences identified in study of *A. cantonensis* lungworm detection in rats in Madagascar, 2022–2023. Red text indicates black rat oral swab specimens. GenBank accession numbers are indicated. For deposited sequences with available metadata, the host species and geolocation of the sample collected is included in parentheses. The tree was rooted with a representative mitogenome of *Parafilaroides normani.*

We detected 5 complete *A. cantonensis* mitochondrial genomes from black rat oral swab specimens, de novo assembled with high coverage (GenBank accession nos. PX571103–7) (Figure 2). All 5 mitochondrial genomes were 13,503 nt in length with 26.8% guanine and cytosine content, in comparison with published *A. cantonensis* mitogenomes that were 13,497–13,519 nt in length with 26.7%–26.8% guanine and cytosine content. The mitogenomes from Madagascar revealed 99.96%–100% sequence similarity among themselves and 96.44%–99.99% similarity compared with global sequences. Of note, the genomes from Madagascar demonstrated >99.98% similarity to sequences from Valencia, Spain (GenBank accession no. PP748572). Phylogenetic analysis placed all sequences from Madagascar within clade II, grouping with the Val-II haplotype and clustering with the haplotype Ac8 from Brazil (Figure 3) (*14*).

*A. cantonensis* prevalence in black rat oral swabs was determined to be 10.4% (95% CI 6.2%–16.9%; 13/125 samples). All positive rats were from the littoral forest of the MSR (Figure 1, panel B). Positive cases were detected in both sampling years, with no significant difference in prevalence between years: 9.67% (95% CI 4.5%–19.6%; 6/62) in 2022 and 11.11% (95% CI 5.5%–21.4%; 7/63) in 2023. Logistic regression analysis identified age as the only significant predictor of infection. Young rats had significantly lower odds of infection compared to adults (odds ratio 0.13, 95% CI 0.02–0.77; p = 0.03) (Table). We observed no significant associations for sex, body condition, weight, or year of sampling (Table).

**Table:** Multivariable logistic regression analysis of factors associated with *Angiostrongylus cantonensis* detection in *Rattus rattus* captured in the littoral forest in southeastern Madagascar, 2022–2023*

Variable	Odds ratio (95% CI)	p value
Age		
Adult	Referent	
Young	0.13 (0.02–0.77)	**0.03**
Sex		
F	Referent	
M	1.13 (0.29–4.59)	0.85
Body condition, continuous	1.97 (0.62–7.07)	0.26
Weight, continuous	0.96 (0.93–1.00)	0.06
Year		
2022	Referent	
2023	0.46 (0.09–1.88)	0.26

*Bold indicates statistically significant value.

## Conclusions

The 5 mitogenomes we obtained were nearly identical to each other (99.96%–100% similarity), indicating a highly conserved parasite population within the MSR littoral forest and low genetic diversity of *A. cantonensis* lungworm in Madagascar, which is similar to another report (*3*). Phylogenetic analysis further demonstrated that all the sequences cluster within clade II and specifically group with the Val-II haplotype, previously reported in Europe and associated with haplogroup Ac8 from Brazil (*14*). That pattern supports the hypothesis of recent or historical introductions mediated by human activities, particularly through the movement of invasive rats and intermediate hosts via trade and transport networks (*3*,*14*).

The overall prevalence in rats in the current study was 10.4% (95% CI 6.2%–16.9%; 13/125 rats), higher than the 2.7% prevalence previously reported in rats from central-eastern Madagascar (n = 78) (*9*). All positive rats were from the littoral forests. Host-related factors also contributed to infection patterns. Age was the only significant predictor; young rats showed markedly lower odds of infection compared with adults. That finding reflects cumulative exposure over time, because older rats have more opportunities to encounter infected intermediate or paratenic hosts (*15*). We observed no significant effects for sex, body condition, weight, or year, suggesting that exposure-driven processes might be more necessary than intrinsic host factors in shaping infection risk. No positive detections were observed in the lowland rainforest or village-edge habitats, potentially because of intermediate host distribution (*5*).

This study provides metagenomic-based mitochondrial genome characterization and prevalence estimates of *A. cantonensis* lungworm from black rats in southeastern Madagascar. Because of the parasite’s life cycle, oral swab specimens likely capture first-stage larvae expelled from the respiratory tract, which are known to be present in the oral cavity during patent infections. However, because necropsy data were not systematically collected for parasitologic examination, we could not assess the relationship between larval detection in oral swab specimens and adult worm burden in the pulmonary vasculature. Future studies combining oral swab specimens with necropsy or quantitative parasitologic methods would help validate this relationship. Nevertheless, our findings demonstrate that complete *A. cantonensis* mitochondrial genomes can be recovered from oral swab specimens, supporting their use as a complementary, minimally invasive approach for molecular detection and genomic characterization of this parasite.
